# Mesenchymal stem cell-derived extracellular vesicles: novel frontiers in regenerative medicine

**DOI:** 10.1186/s13287-018-0791-7

**Published:** 2018-03-09

**Authors:** Somayeh Keshtkar, Negar Azarpira, Mohammad Hossein Ghahremani

**Affiliations:** 1 Department of Molecular Medicine, School of Advanced Technologies in Medicine, Tehran University of Medical Sciences, Tehran, Iran; 2 Transplant Research Center, Shiraz University of Medical Sciences, Shiraz, Iran; 3 Shiraz Institute of Stem Cell and Regenerative Medicine, Shiraz University of Medical Sciences, Shiraz, Iran; 4 Department of Pharmacology-Toxicology, Faculty of Pharmacy, Tehran University of Medical Sciences, Tehran, Iran

**Keywords:** Extracellular vesicles, Mesenchymal stem cells, Regenerative medicine

## Abstract

Mesenchymal stem cells (MSCs) are multipotent stem cells that have gained significant attention in the field of regenerative medicine. The differentiation potential along with paracrine properties of MSCs have made them a key option for tissue repair. The paracrine functions of MSCs are applied through secreting soluble factors and releasing extracellular vesicles like exosomes and microvesicles. Extracellular vesicles are predominantly endosomal in origin and contain a cargo of miRNA, mRNA, and proteins that are transferred from their original cells to target cells. Recently it has emerged that extracellular vesicles alone are responsible for the therapeutic effect of MSCs in plenty of animal diseases models. Hence, MSC-derived extracellular vesicles may be used as an alternative MSC-based therapy in regenerative medicine. In this review we discuss MSC-derived extracellular vesicles and their therapeutic potential in various diseases.

## Background

Progress in the field of regenerative medicine is occurring through a variety of approaches for the repair of damaged tissues or lost cells. One recent approach is to use stem cells, including mesenchymal stem cells (MSCs). Several studies have shown that MSCs can play an influential role in the regeneration of injured tissues and cells in various diseases via differentiation or the secretion of beneficial factors and vesicles [1, 2]. Recent research has focused on vesicles secreted by MSCs as a possible non-cellular therapy [3]. Accordingly, this review describes the vesicles released by MSCs and their effects on different disease models.

## Mesenchymal stem cells

MSCs are described as multipotent nonhematopoietic adult stem cells that express the surface markers CD90, CD105, and CD73, without the expression of CD14, CD34, and CD45 [4]. They were originally found by Friedenstein [4] via studies on the bone marrow in the 1960s but can be isolated from other adult tissues, such as adipose tissue, dental pulp, placenta, amniotic fluid, umbilical cord blood, Wharton’s jelly, and even the brain, spleen, liver, kidney, lung, thymus, and pancreas [4, 5]. MSCs can adhere to plastic surfaces and simply extend ex vivo [6].

MSCs have various unique features, including differentiation potential and colony forming and self-renewal abilities [7]. They can be differentiated into mesenchymal lineages, namely osteoblasts, chondrocytes, adipocytes, endothelial cells, and cardiomyocytes, as well as non-mesenchymal lineages, such as hepatocytes, and neuronal cell types [6]. Besides their differentiation potential, MSCs have the ability to secrete some trophic factors such as growth factors, cytokines, etc. [8].

In recent years MSCs have appeared as a promising approach for regeneration of various tissues [9]. It was originally thought that MSCs exert their therapeutic effect by migrating to sites of damage, engrafting, and subsequently differentiating into desired cells for tissue regeneration. However, other studies have indicated that the therapeutic benefit of MSCs is attributable not only to their differentiation but also through factors they secrete [8].

## Paracrine action of MSCs

Paracrine secretion by MSCs was first identified by Haynesworth et al. [10]. They reported that MSCs produce and release a broad repertoire of growth factors, chemokines, and cytokines that modulate the action of adjacent cells. In fact, these secreted factors increase angiogenesis, reduce apoptosis and fibrosis, enhance neuronal survival and differentiation, stimulate extracellular matrix remodeling, restrict local inflammation, and adjust immune responses. In this way, MSCs directly or through paracrine secretion induce regeneration for rescuing injured cells, decreasing tissue injury, and finally accelerating organ repair [2, 4, 11].

Several studies have investigated the therapeutic effects of MSC-derived paracrine factors on different disorders, including bone and cartilage regeneration in immune diseases, neurological diseases, liver injury, acute kidney failure, and cardiovascular diseases [12].

These studies have indicated that molecules secreted by MSCs perform an effective role as mediators which either directly activate the target cells or stimulate neighboring cells to secrete active factors [2]. Recently, however, it has been recognized that MSCs release numerous extracellular vesicles (EVs) that participate in tissue regeneration via transferring information to damaged cells or tissue and exert biological activity similar to the MSCs [3].

## Extracellular vesicles

The secretion of EVs during maturation of reticulocytes was recognized in 1983 [13]. EVs are membrane-packed vesicles that are secreted by a variety of cell types, including T cells, B cells, dendritic cells, platelets, mast cells, epithelial cells, endothelial cells, neuronal cells, cancerous cells, oligodendrocytes, Schwann cells, embryonic cells, and MSCs [14].

EVs can also be found in physiological fluids such as normal urine, blood, bronchial lavage fluid, breast milk, saliva, cerebrospinal fluid, amniotic fluid, synovial fluid, and malignant ascites. The most important EVs are microvesicles (MVs) and exosomes [13, 14]. It has been demonstrated that EVs perform an important role in cell-to-cell communication. They have been implicated in important processes such as immune responses, homeostasis maintenance, coagulation, inflammation, cancer progression, angiogenesis, and antigen presentation. Thus, EVs participate in both physiological and pathological conditions [13, 14].

### Main classes of EVs

#### Exosomes

**Fig. 1:**
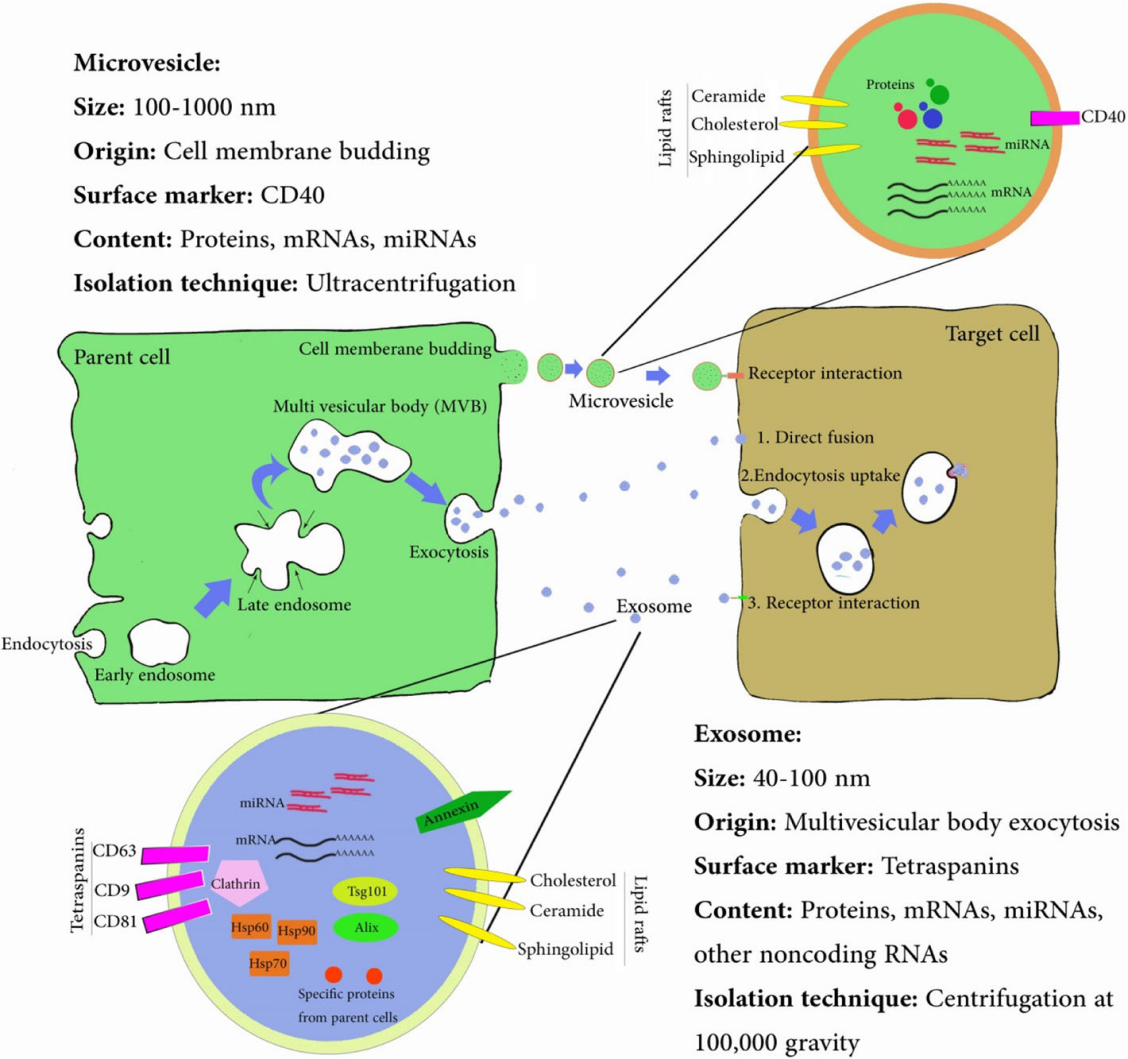
Origin, content, and intercellular communication of extracellular vesicles. Hsp heat shock protein, MVB multivesicular body, Tsg 101 tumor susceptibility gene 101

Exosomes comprise one of the main subclasses of EVs and have an endosomal origin [15]. The biogenesis of exosomes occurs via the endocytosis-exocytosis pathway when cells absorb small amounts of intracellular fluid in a specific membrane region and form early endosomes. The early endosome begins to mature and expands into a late endosome; then intraluminal vesicles or multivesicular bodies (MVBs) are formed by internal budding of the endosomal membrane. The MVBs then fuse to the cell membrane and are released into the extracellular environment. At this point the vesicles are named exosomes (Fig. 1) [14], which are released via exocytosis that is regulated by p53 and under the control of the cytoskeleton activation pathway but not affected by calcium [8, 13]. Exosomes have a diameter of 40–100 nm and a density of 1.13 to 1.19 g/mL in a sucrose gradient; they can be collected by centrifugation at 100,000 *g*. After isolation, they can be stored without any toxic cryoprotectant agents at −80 °C for more than 6 months while maintaining their functions [16]. Exosomes contain large amounts of annexins, tetraspanins such as CD63, CD81, and CD9, and heat-shock proteins, including Hsp60, Hsp70, and Hsp90. They also express Alix, tumor susceptibility gene 101 (Tsg101), and clathrin. Exosomes are encapsulated in a bilayer membrane that protects their contents and enables them to move long distances in tissues. The membrane possesses small amounts of phosphatidylserine but large amounts of cholesterol, ceramide, and sphingolipids [13, 16]. Exosomes contain a cargo of genetic materials (mRNA, miRNA, pre-miRNA, and other noncoding RNA) and proteins that are transferred to and released into target cells [13, 14]. When exosomes are released into the extracellular environment, they can interact with recipient cells via three pathways. They may enter cells via endocytic uptake or by direct fusion of the vesicles to the cell membrane. They may also transmit their contents through adhesion to the cell surface mediated by the interaction of a lipid–ligand receptor (Fig. 1). These interactions indicate that exosomes possess pivotal roles in cell-to-cell communication and immune modulation in different physiologic and pathologic conditions [13, 15, 17].

Exosomes have also been studied in degenerative diseases. It was shown that neurons from Parkinson’s and Alzheimer patients secrete exosomes containing alpha-synuclein and amyloid beta protein that are, respectively, the hallmarks of progression of these diseases [5].

#### Microvesicles

MVs, or shedding vesicles, are formed by external budding of the cell membrane in different cell types that involves cytoskeleton reorganization and is also dependent on the concentration of intracellular calcium [13]. They have a diameter of 100–1000 nm and can be isolated by ultracentrifugation with a density of 1.04 to 1.07 g/mL in a sucrose gradient [3]. MVs contain high amounts of phosphatidylserine-containing proteins associated with lipid rafts and are rich in the surface marker CD40 as well as cholesterol, sphingomyelin, and ceramide. They carry a cargo of proteins, lipids, mRNAs, and microRNAs and interact with recipient cells by specific receptor–ligand interactions (Fig. 1) [14].

MVs may change functional target cells by delivering intracellular proteins; for example, MVs released from endothelial cells can promote angiogenesis through transfer of proangiogenic molecules such as growth factors and their activator [12]. They may also horizontally transfer genetic information to target cells [18].

## Therapeutic effects of EVs derived from MSCs

As described earlier, MSCs have attracted the attention of researchers in the field of regenerative medicine due to their ease of isolation from different and accessible adult tissues and their ability to be cultured in vitro. Initially the differentiation potential of MSCs was considered as the cell replacement strategy [10].

In recent years, however, focus on the therapeutic effects of MSCs in regenerative medicine has been shifting to their activity through paracrine secretion instead of engraftment and differentiation into functional cells [2, 4].

In fact, MSC-derived EVs (MSC-EVs), or cell-free therapies, in contrast to treatments based on whole cells, are easier to manage and safer due to lower amounts of membrane-bound proteins such as MHC molecules and their inability to directly form tumors [19].

**Fig. 2:**
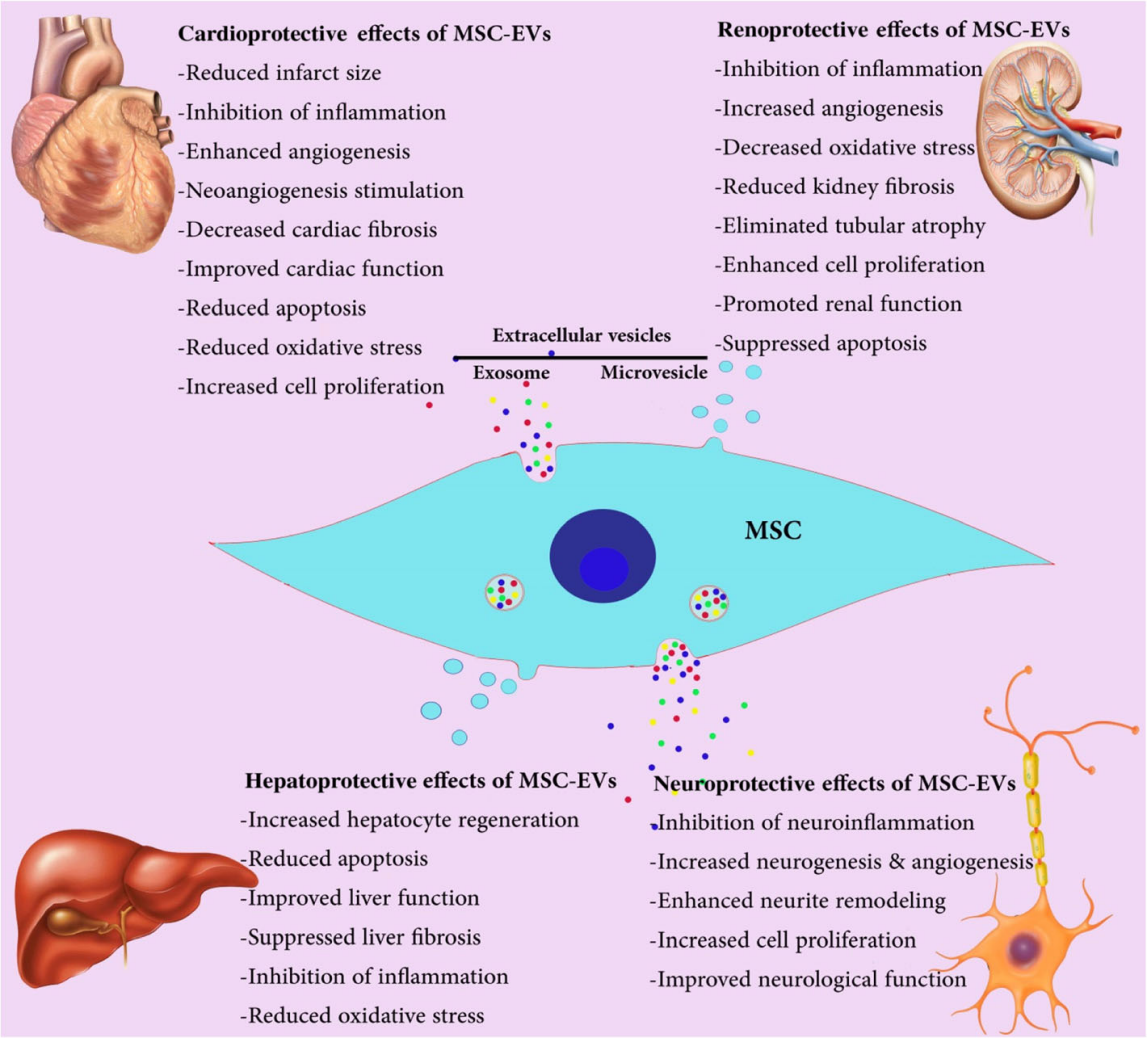
Therapeutic effects of mesenchymal stem cell-derived extracellular vesicles (MSC-EVs) in kidney, liver, cardiovascular, and neurological diseases

MSC-EVs were first studied in 2010 by Lai et al. [20] in a mouse model of myocardial ischemia-reperfusion (MI/R) injury and were thereafter investigated in several disease models. We summarize the information form recent research on MSC- EVs in kidney liver cardiovascular and neurological diseases in the following sections (Fig. 2)

.

### MSC-derived EVs in kidney diseases

Several studies have reported that MSC-EVs possess renoprotective effects in kidney injury models. Bruno et al. [21] tested the effect of human bone marrow MSC-EVs in glycerol-induced AKI in SCID mice. They reported that MSC-EVs improved tubular injury and renal function by inducing tubular cells for proliferation. Their results suggest that activation of the proliferative pathway in tubular cells may occur via horizontal transfer of mRNAs by MSC-EVs . In another glycerol-induced AKI mouse model the prominent role of the contents of MSC-EVs was studied. MSC-EVs exerted proliferative and anti-apoptotic effects on tubular epithelial cells by transferring specific mRNAs and miRNAs and growth factors that influence cell cycle entry and progression and regulate proliferative/anti-apoptotic pathways, leading to restoration of the kidney [22].

In another study He et al. [23] directly compared the effects of MSCs and MSC-EVs in a nephrectomy mouse model and showed that, similar to MSCs, MSC-EVs reduced fibrosis and decreased or eliminated tubular atrophy in the treated group compared with the untreated group. This study indicated that MSC-EVs might replicate the effective behavior of MSCs.

The effects of MSC-EVs were investigated in AKI induced by cisplatin in SCID mice. A single injection of MSC-EVs improved survival by ameliorating the affected renal function and morphology but was not able to prevent chronic tubular injury. However, multiple injections of EVs further reduced mortality [24]. An in vitro study by the same group showed that EVs increased the expression of anti-apoptotic genes such as Bcl-xL, Bcl2, and BIRC8 and decreased the expression of proapoptosis genes such as Caspase1, Caspase 8, and lymphotoxin alpha [24].

In a rat model of kidney ischemia reperfusion injury (IRI) Gatti et al. [25] showed that injection of MSC-EVs protected rats from AKI through reducing apoptosis and increasing tubular epithelial cell proliferation and subsequently improved renal function. The renoprotective effect was abolished after RNase pretreatment of EVs, suggesting EVs could protect tubular cells via transferring effective mRNA and miRNA to recipient cells.

In vitro studies also emphasized the role of horizontal transfer of miRNA and mRNA from MSC-EVs in enhancing renal cell proliferation and reducing cell death. In one study MSC-EVs co-incubated with proximal tubular epithelial cells (PTECs) that had been injured by cisplatin enhanced proliferation through transferring mRNA for receptor of insulin-like growth factor-1 (IGF1-R) [26]. In another study, PTECs damaged by ATP depletion were exposed to MSC-EVs. The EVs were incorporated into the injured PTECs and reduced apoptosis and reinstituted proliferation of the cells. The MSC-EVs transferred miRNA to the PTECs that led to modulation of several miRNAs inside the PTECs and subsequently to downregulation of apoptosis, hypoxia, and cytoskeletal reorganization genes. In fact, the MSC-EVs protected PTECs through regulating post-transcriptional pathways [27].

Moreover, Zhou et al. [28] showed that human umbilical cord MSC-EVs were able to repair cisplatin-induced AKI in rats and an in vitro renal injury by improving oxidative stress, suppressing cell apoptosis, and promoting cell proliferation through activation of the ERK1/2 pathway.

In a recent animal study Ranghino et al. [29] studied the effect of EVs derived from glomerular MSCs on AKI induced by IRI in SCID mice. They demonstrated that glomerular MSC-EVs ameliorated the affected kidney function and reduced renal injury after IRI via tubular epithelial cell proliferation mediated by mRNAs and miRNAs transferred by the MSC-EVs.

Studies have shown that the renoprotective effects of MSC-EVs partly relate to the pro-angiogenesis effect of EVs. Choi et al. [30] investigated the effect of EVs derived from kidney MSCs on AKI induced by IRI in a mouse model. The results showed EVs shuttled several pro-angiogenic transcription factors including VEGF, IGF-1, and bFGF, which led to renal cell recovery and overall improvement of kidney function.

In another study Zou et al. [31] explored the pro-angiogenesis effect of MSC-EVs in a rat model of kidney IRI. They observed that EVs could reduce cell apoptosis and enhance proliferation, and subsequently improved renal function and decreased the histological lesion via transferring pro-angiogenesis genes such as VEGF. In a similar rat model of kidney IRI the delivery of EVs derived from umbilical cord MSCs improved kidney function [32]. The EVs transferred pro-angiogenic RNAs which induced injured tubular cells to express HGF mRNA that was translated to HGF protein, thus boosting tubular cell regeneration [32].

MSC-EVs are also renoprotective via their anti-inflammatory effects. In an IRI rat model injection of human Wharton’s jelly-derived MSC-EVs reduced the number of macrophages in the damaged kidney and suppressed CX3CL1 expression. Thereby the EVs improved renal injury in the acute and chronic stages via their anti-inflammatory properties [33]. In a similar study delivery of adipose-derived MSC-EVs in rats with renal IRI promoted renal function by reducing expression of inflammatory cytokines such as TNFα and IL-1β [34].

Furthermore, injection of MSC-EVs in rats with AKI induced with gentamycin reduced the amount of pro-inflammatory cytokines such as IL-6 and TNF-α and enhanced the level of IL-10, which has an anti-inflammatory effect. This treatment boosted renal cell proliferation and suppressed apoptosis and necrosis [35].

It has also been demonstrated that injection of MSC-EVs in an AKI rat model induced with cisplatin promoted kidney function by activating autophagy-related genes, including ATG5 and ATG7, and enhancing expression of the autophagy marker protein LC3B in parallel with suppressing apoptosis and inflammatory cytokine secretion [36].

Further studies have shown that the renoprotective effects of MSC-EVs are partly related to their anti-oxidant effects. Zhang et al. [37] reported that a single injection of MSC-EVs suppressed expression of NADPH oxidase and production of reactive oxygen species and thus reduced oxidative stress in the early stage of renal injury in an IRI rat model, thereby alleviating fibrosis and promoting kidney function.

Another study investigated the role of the anti-oxidative properties of MSC-EVs in an AKI rat model [38]. The results showed that MSC-EVs reduced renal tubular injury, improved renal function, and decreased apoptosis. In addition, MSC-EVs reduced oxidative stress in renal injury via increasing Nrf2/anti-oxidant response element (ARE) activity. Nrf2 is the main regulator of anti-oxidant responses connected to AREs, which activate the expression of anti-oxidant genes. So MSC-EVs may protect against AKI also through the anti-oxidative pathway [38].

### MSC-derived EVs in liver diseases

Some studies have reported that MSC-EVs can be used for treatment of liver disease in animal models. Li et al. [39] studied the effect of human umbilical cord MSC-EVs in a fibrotic liver mouse model induced by carbon tetrachloride (CCl_4_). They demonstrated that transplantation of MSC-EVs could improve liver fibrosis and protect hepatocytes via suppression of epithelial-to-mesenchymal transition and inactivation of the TGF-β1/Smad 2 pathway [39]. In other study Tan et al. [40] investigated the effect of MSC-EVs on an in vitro model of acetaminophen or H_2_O_2_-induced hepatocyte injury and in a mouse model of CCl4-induced acute liver injury. They showed that MSC-EVs increased hepatocyte regeneration by up-regulation of proliferation proteins such as PCNA and Cyclin D1 and the anti-apoptosis gene Bcl-xL.

MicroRNAs associated with MSC-EVs also have important effects in liver protection. For example, Hyun et al. [41] studied the effect of chorionic plate-derived MSC-EVs containing miR-125b on CCl4-induced liver fibrosis in a mouse model. They found that EVs containing miR-125b improved hepatic fibrosis by suppressing the activation of Hedgehog (Hh) signaling through the blockade of Smo expression.

Another study demonstrated that adipose tissue-derived MSC-EVs expressing miR-122 led to decreased proliferation and activation of hepatic stellate cells (HSCs) in a liver fibrosis model. Furthermore, MSC-EVs containing miR-122 could stimulate the expression of miR-122 target genes such as IGF1-R, CCNG1, and P4HA1 in HSCs. These target genes are involved in proliferation and maturation of collagen [42].

Moreover, Tamura et al. [43] evaluated the effect of MSC-EVs on concanavalin-A-induced liver injury as an immune-induced liver injury model. They showed that EVs reduced the level of serum alanine aminotransferase (ALT) and decreased production of proinflammatory cytokines, while anti-inflammatory cytokine and regulatory T cell levels increased, suggesting that MSC-EVs have an anti-inflammatory effect.

Likewise the anti-oxidant and anti-apoptotic effects of MSC-EVs were investigated in an acute liver injury mouse model. EVs derived from human umbilical cord MSCs shuttled glutathione peroxidase 1 (GPX1), an important human anti-oxidant. A single injection of MSC-EVs significantly rescued the recipient mice from acute liver injury through detoxifying hydrogen peroxide and decreasing oxidative stress and cell death by GPX1 [44].

Recently Haga et al. [45] studied the effect of MSC-EVs on a lethal mouse model of liver failure induced by D-galactosamine/TNF-α. The results showed that MSC-EVs mitigated hepatic injury and enhanced survival through modulating the inflammatory response and activating anti-apoptotic pathways. In fact, MSC-EVs carried specific RNAs, including Y-RNA-1, that show protective effects on hepatocyte mortality, suggesting that EVs have hepatoprotective effects via shuttling effective genetic information from MSCs.

### MSC-derived EVs in cardiovascular diseases

Lai et al. [20] reported that MSC-EVs are cardioprotective. They demonstrated that human embryonic stem cell MSC-EVs reduced infarct size in a mouse model of MI/R injury. Subsequently, another study from the same group showed that MSC-EV treatment in a myocardial infarction (MI) mouse model led to decreased infarct size, enhanced NADH and ATP levels, and reduced oxidative stress. Also, the MSC-EV treatment increased phosphorylation of Akt and GSK-3β (the anti-apoptotic pathway), and reduced phosphorylation of c-JNK (the proapoptotic pathway) in I/R myocardium. These results were in parallel with decreasing macrophage and neutrophil infiltration in the heart after MI/R. Hence, treatment by MSC-EVs could improve cardiac function through decreased oxidative stress and activation of pro-survival signaling in a MI/R injury model [46]. It has also been shown that MSC-EVs improved cardiac function through stimulation of neoangiogenesis and suppression of the inflammation response in both in vitro and in vivo MI models [47, 48].

MicroRNAs associated with MSC-EVs are also important in cardioprotection. For instance, Feng et al. [49] found that EVs enriched with miR-22 which were released by MSCs could reduce cardiac apoptosis and fibrosis via downregulation of methyl CpG binding protein 2 (Mecp2) in an acute myocardial infarction (AMI) mouse model. Furthermore, Yu et al. [50] showed that MSC-EVs containing miR-221 displayed anti-apoptotic effects in an in vitro ischemic heart injury model. EVs increased cardioprotection by reducing the expression of PUMA (p53 upregulated modulator of apoptosis).

Another study by the same group demonstrated that MSC-EVs enriched with miR-19a restored cardiac function and reduced infarct size in a rat model of AMI. In fact, miR-19a exerted cardioprotective effects through downregulation of phosphatase and tensin homolog (PTEN) and subsequent activation of the Akt and ERK signaling pathways [51]. MSC-EVs enriched with miR-210 also ameliorated cardiac function via enhancing of angiogenesis in both in vitro and in vivo MI models [52]. Furthermore the effects of endometrium-derived MSC-EVs were investigated in a rat MI model and it was clear that these EVs shuttle miR-21 [53]. MSC-EVs restored cardiac function and decreased infarct size via miR-21-mediated PTEN downregulation and AKt activation. Finally, the pro-survival gene Bcl-2 and VEGF were upregulated.

In a remarkable study Zhang et al. [54] preconditioned cardiac stem cells (CSCs) with MSC-EVs and then transplanted the CSCs into a rat MI model. The pretreatment of CSCs with MSC-EVs decreased cardiac fibrosis and increased survival and capillary density, and in this way improved cardiac function. The other beneficial effect of MSC-EVs in a mouse model of hypoxic pulmonary hypertension was studied by Lee et al. [55]. They showed that MSC-EVs reduced progression of pulmonary hypertension and right ventricular hypertrophy, possibly through inhibition of STAT3 signaling.

Altogether, these studies imply the key role of MSC-EVs in improving cardiac function and morphology is the transfer of their specific cargos with anti-apoptotic, anti-inflammatory, anti-oxidant, and pro-survival effects.

### MSC-derived EVs in neurological diseases

MSC-EVs have shown potential therapeutic benefit in the treatment of neurological and neurodegenerative diseases. Xin et al. studied the effect of MSC-EVs in several studies [56–58]. The intravenous administration of MSC-EVs improved neurological recovery and neurovascular plasticity in a rat stroke model [56]. They also demonstrated that MSC-EVs through transfer of miR-133b to neuron cells and astrocytes enhanced neurite remodeling and improved functional recovery in the rat stroke model [57]. Xin et al. also reported that MSC-EVs enriched with the miR-17-92 cluster enhanced oligodendrogenesis neurogenesis neural plasticity and functional recovery after stroke possibly by suppressing PTEN and subsequently by increasing the phosphorylation of proteins downstream of PTEN including of the protein kinase B/mechanistic target of rapamycin/glycogen synthase kinase 3β signaling pathway [58].

Similarly, Doeppner et al. [59] found that MSC-EVs induced long-term neuroprotection, improved neurological recovery, increased neurogenesis and angiogenesis, and adjusted post-ischemic immune responses in a mouse stroke model. It has also been shown that MSC-EVs improved brain function by reducing the neurological sequelae in a preclinical model of preterm hypoxic-ischemic brain injury [60]. In another study, the effects of MSC-EVs were investigated in a rat model of preterm brain injury [61]. Interestingly, EVs improved inflammation-induced neuronal cellular degeneration, decreased microgliosis, and inhibited reactive astrogliosis. Moreover, treatment with MSC-EVs promoted long-lasting cognitive functions [61].

MSC-EVs have also been tested in traumatic brain injury (TBI) models. The results indicated that MSC-EVs increased angiogenesis and neurogenesis reduced neuroinflammation enhanced newly generated endothelial cells and newly formed immature and mature neurons and thereby improved functional recovery after TBI [62]. In another TBI mouse model MSC-EV treatment inhibited neuro-inflammation after TBI and salvaged pattern separation and spatial learning impairments [63].

The impact of MSC-EVs was investigated in Alzheimer’s disease by Katsuda et al. [64]. They used EVs secreted from adipose tissue MSCs. These EVs contain large amounts of neprilysin, the most prominent enzyme that degrades β-amyloid peptide in the brain. Transfer of EVs into neuroblastoma cells led to reductions in both secreted and intracellular β-amyloid peptide levels, which might be a therapeutic approach to Alzheimer’s disease. Jarmalavičiūtė et al. [65] studied the effect of EVs secreted by dental pulp-derived MSCs on dopaminergic neurons in a 3D culture. The data demonstrated that these EVs rescued dopaminergic neurons from 6-hydroxy-dopamine-induced apoptosis and presumably could be used as a treatment for Parkinson’s disease in the future.

## Conclusions

MSC-EVs, especially exosomes have gained significant interest with regard to their use as regenerative therapies. EVs can be readily isolated from MSCs of various origin and carry biologically active molecules which can be transferred to target cells to exert their therapeutic effects like regenerating tissue injuries suppressing inflammatory responses modulating the immune system and many other beneficial effects. Accordingly EVs could be an effective safe and cheap therapeutic approach in cell-free regenerative medicine and might become a suitable alternative instead of MSCs. However detail about the functional mechanisms of EVs is not clear and needs to be determined to take full advantage of MSC-EVs in regenerative therapy. As the next steps effort should be directed toward achieving standard methods for EV isolation characterization and administration to provide effective safe and powerful new therapies based on MSC-EVs.

## Abbreviations

AKI: Acute kidney injury
AMI: Acute myocardial infarction
ARE: Antioxidant response element
bFGF: Basic fibroblast growth factor
CSC: Cardiac stem cell
EV: Extracellular vesicle
HGF: Hepatocyte growth factor
HSC: Hepatic stellate cell
Hsp: Heat-shock protein
IGF-1: Insulin-like growth factor 1
IGF1-R: Insulin-like growth factor1-receptor
IL: Interleukin
I/R: Ischemia/reperfusion
IRI: Ischemia reperfusion injury
MI: Myocardial infarction
MI/R: Myocardial ischemia-reperfusion
miRNA: MicroRNA
MSC: Mesenchymal stem cell
MSC-EV: MSC-derived EV
MVB: Multivesicular body
MV: Microvesicle
PTEC: Proximal tubular epithelial cell
PTEN: Phosphatase and tensin homolog
TBI: Traumatic brain injury
TGF: Transforming growth factor
TNF: Tumor necrosis factor
VEGF: Vascular endothelial growth factor
